# A large, population-based study of age-related associations between vaginal pH and human papillomavirus infection

**DOI:** 10.1186/1471-2334-12-33

**Published:** 2012-02-08

**Authors:** Megan A Clarke, Ana Cecilia Rodriguez, Julia C Gage, Rolando Herrero, Allan Hildesheim, Sholom Wacholder, Robert Burk, Mark Schiffman

**Affiliations:** 1 Division of Cancer Epidemiology and Genetics, DHHS, National Cancer Institute, National Institutes of Health, Bethesda, MD, USA; 2 Proyecto Epidemiológico Guanacaste, San José, Costa Rica; 3 Department of Pediatrics, Microbiology & Immunology; Epidemiology & Population Health; and Obstetrics, Gynecology, and Women's Health, Albert Einstein College of Medicine, Bronx, NY, USA; 4 Clinical Genetics Branch, Division of Cancer Epidemiology and Genetics (DCEG), National Cancer Institute, 6120 Executive Blvd, EPS/7011, Rockville, MD 20852, USA

**Keywords:** HPV, Vaginal pH, Cervical neoplasia, Aging, Chlamydia

## Abstract

**Background:**

Vaginal pH is related to genital tract inflammation and changes in the bacterial flora, both suggested cofactors for persistence of human papillomavirus (HPV) infection. To evaluate the relationship between vaginal pH and HPV, we analyzed data from our large population-based study in Guanacaste, Costa Rica. We examined vaginal pH and the risk of HPV infection, cytological abnormalities, and *C. trachomatis *infection.

**Methods:**

Our study included 9,165 women aged 18-97 at enrollment with a total of 28,915 visits (mean length of follow-up = 3.4 years). Generalized estimating equations were used to evaluate the relationship between vaginal pH and HPV infection (both overall and single versus multiple types) and low-grade squamous intraepithelial lesions (LSIL), the cytomorphic manifestation of HPV infection. The relationship between enrollment vaginal pH and *C. trachomatis *infection was assessed by logistic regression. Results were stratified by age at visit.

**Results:**

Detection of HPV was positively associated with vaginal pH, mainly in women < 35 years (p-trend = 0.009 and 0.007 for women aged < 25 and 25-34 years, respectively). Elevated vaginal pH was associated with 30% greater risk of infection with multiple HPV types and with LSIL, predominantly in women younger than 35 and 65+ years of age. Detection of *C. trachomatis *DNA was associated with increased vaginal pH in women < 25 years (OR 2.2 95% CI 1.0-5.0).

**Conclusions:**

Our findings suggest a possible association of the cervical microenvironment as a modifier of HPV natural history in the development of cervical precancer and cancer. Future research should include studies of vaginal pH in a more complex assessment of hormonal changes and the cervicovaginal microbiome as they relate to the natural history of cervical neoplasia.

## Background

The development of cervical cancer is linked to persistent infection with at least one of about a dozen carcinogenic genotypes of human papillomavirus (HPV)[1]. Most HPV infections are transient [2] and only a small minority of women with long-term, persistent infections are at an increased risk of progression to precancerous cervical lesions and cervical cancer [2,3]. Although this causal role is well established, relatively little is known about non-behavioral factors that influence risk of HPV acquisition and persistence. Alterations in the vaginal microenvironment, due to vaginal douching [4], bacterial vaginosis (BV) [5], and sexually transmitted infections [6,7] have been implicated as cofactors for persistence of HPV infection. Although the mechanisms for these associations, if ultimately confirmed, are not completely understood, factors relating to the vaginal microenvironment such as an acidic vaginal pH and the presence of *Lactobacilli *are key components of the vaginal defense system [8]. Changes in vaginal microflora such as bacterial vaginosis, and vaginal infections are usually accompanied by increases in pH [9,10] while acidic vaginal pH has been associated with decreased risk of *C. trachomatis*, trichomoniasis [11], urinary tract infections [12], and mycoplasma [11]. Therefore, it is conceivable that vaginal pH could be associated with HPV prevalence, through unknown direct or indirect mechanisms affecting acquisition or persistence.

Vaginal pH reflects a combination of factors affecting the vaginal microenvironment. In healthy, reproductive aged women, vaginal pH is primarily determined by the lactic acid produced by the metabolically active epithelium and from *Lactobacillus *species that dominate the vaginal microflora and produce lactic acid from anaerobic glycolysis [8]. Both processes are fueled by glucose which is stored as glycogen in the vaginal mucosal cells [8]. A pH range of 4.0-4.5 is considered normal for pre-menopausal women [13]. After menopause the pH increases, as circulating estrogens decline and cause a depletion of glycogen and glucose metabolism, and a loss of *Lactobacilli *in the vaginal mucosa [8]. It has been theorized that this rise in vaginal pH is associated with a loss of natural epithelial defenses and an increased rate of colonization with pathogens in the vagina and the urinary tract [14].

Our study examined the relationship between vaginal pH and risk of HPV infection and HPV-related cytological abnormalities, with particular attention to possible differences between women in varying age groups in a large screening study of HPV infection and cervical neoplasia in Guanacaste Province, Costa Rica. In addition, we evaluated whether there is an association between increased vaginal pH and *C. trachomatis *DNA measured from cervical cells obtained in a random subset of cohort members at enrollment.

## Methods

### Study population

**Figure 1:**
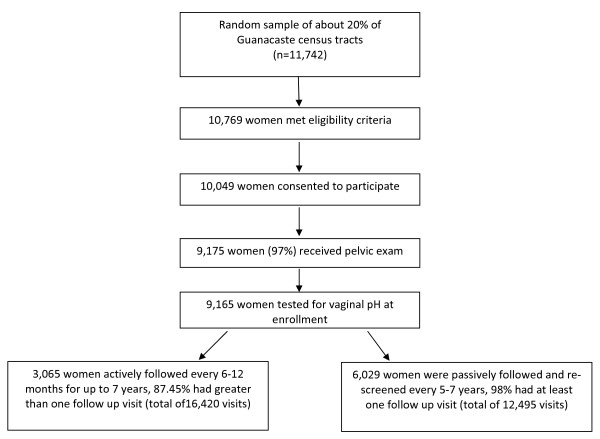
**Consort diagram**.

Vaginal pH was measured during enrollment and follow-up screening visits of a population-based cohort study of cervical neoplasia in Guanacaste, Costa Rica. Detailed methods of recruitment, screening, and follow-up have been reported elsewhere [15,16]. Briefly, a random sample of about 20% of Guanacaste census tracts identified 11,742 women, of whom 10,769 were eligible for the study (Figure 1). Of these eligible women, 10,049 agreed to participate and were interviewed after giving informed consent. A pelvic examination was offered to women who reported previous sexual intercourse in the interview and of the 9,466 eligible, 9,175 (97%) had the exam performed. At enrollment, vaginal pH measurements were obtained from 9,165 women. A subcohort of women (n = 3,065) who were at risk of developing CIN3 or cancer (CIN3+) based on enrollment screening results and reported sexual behavior, as well as a group of women who had negative screening results (referent group) were actively followed at intervals of 6 or 12 months for up to 7 years. The remaining 6,029 women, who were considered (and subsequently proven) to be at very low risk of cervical cancer based on completely negative multimodal screening, were included in the passive follow-up cohort that was screened again at 5-7 years after enrollment [16]. Among the 9,094 women followed, 8,080 (88.8%) had at least one follow-up visit. Of the active cohort, 87.4% had greater than one follow up visit whereas 98% of the passive cohort had at least one follow up visit. The median follow-up times in both cohorts were equivalent (6-7 years), although the screening intensity was clearly higher in the active cohort. The study protocol was reviewed and reapproved annually by the National Cancer Institute and a Costa Rican Institutional Review Board.

### Specimen collection

Exfoliated cervical cells for split-sample conventional Pap smear and ThinPrep cytology (Cytyc Corp., Boxborough, MA) were collected using a Cervex broom-like collection device (Unimar, Wilton, CT) during the pelvic exam. Additional cervical cells were collected using a Dacron swab and stored in Specimen Transport medium (Qiagen, previously Digene Corp., Gaithersburg, MD) for HPV testing. Cervical abnormalities were identified by visual inspection, cytology, or cervicography. Cervical cytologic abnormalities were classified as cancer and/or high-grade squamous intraepithelial lesions (HSIL+), low-grade squamous intraepithelial lesions (LSIL), atypical squamous cells of undetermined significance (ASCUS), reactive changes, or normal. As the current analysis focused on HPV-related cytomorphologic changes, a woman's cytology was interpreted as LSIL if her conventional smear and/or ThinPrep results met the criteria for LSIL and the diagnosis of the alternative method was either ASCUS or LSIL.

### HPV determination by polymerase chain reaction

Specimens from the enrollment visit were tested initially in the United States by the Hybrid Capture Tube test (Qiagen, previously Digene Corp., Gaithersburg, MD). However, because this method had limited sensitivity (10 pg/mL) and only detected 11 of the carcinogenic HPV types, all specimens were retested using a polymerase chain reaction (PCR) test. As previously described [17,18], DNA extracted from exfoliated cells in a 100-μL aliquot of the specimen was amplified using the MY09/MY11 L1 degenerate primer PCR system with AmpliTaq Gold polymerase (TaqGold; Perkin-Elmer-Cetus, Norwalk, CT). After amplification, PCR products were analyzed by electrophoresis and hybridized with radiolabeled generic HPV DNA probes. Type specific oligonucleotide hybridization was used for HPV typing: Probes were specific for types 2, 6, 11, 13, 16, 18, 26, 31-35, 39, 40, 42-45, 51-59, 61, 62, 64, 66-74, 81-85, and 89. We considered the following HPV types as carcinogenic: HPV16, 18, 31, 33, 35, 39, 45, 51, 52, 56, 58, and 59 [19]. The HPV testing lab was masked to clinical outcomes. All PCR testing and genotyping were done in the United States, at the Albert Einstein College of Medicine in New York.

### Vaginal pH measurements

Vaginal pH was measured with a pHydrion strip (Micro Essential Laboratories, Brooklyn, NY). After insertion of a sterile speculum the pHydrion strip was placed on the left lateral vaginal wall between the speculum blades until moistened and color change was immediately compared with the colorimetric scale and the measurement was recorded across a range of 3.0-5.5 in increments of 0.5 pH units.

### Detection of *C. trachomatis *DNA

Detection of *C. trachomatis *was measured with an assay for *C. trachomatis *DNA as previously described [20] in an age-stratified subsample (N = 1,216). Briefly, *C. trachomatis *DNA was detected in enrollment samples using a *C. trachomatis *PCR-DEIA assay (Labo Biomedical Products BV, Rijswijk, the Netherlands). After the PCR, *C. trachomatis *DNA was detected by *C. trachomatis*--specific probe hybridization in a DNA enzyme immunoassay that used 10 μL of the PCR product. The mixture of probes present in the *C. trachomatis *DNA enzyme immunoassay can recognize all *C. trachomatis *serovars and genovariants that have been deposited in GenBank.

### Statistical analysis

The unit of analysis was the study visit. Based on their age at each visit, women were stratified into the following categories: < 25 years, 25-34 years, 35-44 years, 45-54 years, 55-64 years and 65+ years. To standardize frequency of visits by time, each clinic visit was assigned to a bin defined by years after enrollment. Bins for each woman began with bin 0 (enrollment date), continued for every year until they were censored (for suspicion of HSIL+ or completion of the follow-up, i.e., at least 6 years and 9 months from enrollment) or they were lost to follow-up. If a woman was tested more than once in a bin and her HPV results were discordant on positivity, the result from the positive visit was taken to acknowledge the possibility of HPV measurement error. Similarly, if pH was tested more than once in a bin, the visit with the highest vaginal pH recorded was chosen. Regarding cytology, if more than one result was available, the more severe reading was chosen.

Our main analyses focused on risk of HPV positivity with vaginal pH as the primary independent variable. We calculated the odds of testing HPV positive vs. testing negative and the odds of testing positive for multiple HPV types vs. a single HPV type. For all of the above-mentioned analyses, we used the method of generalized estimating equations (GEE) with an independent correlation structure [21]. This approach accounted for repeated visits resulting in possible auto-correlation and reduced overall variance, but it made few assumptions regarding the nature of the intra-individual correlations [22]. To determine if elevated vaginal pH was also related to HPV-induced cytological abnormalities, we calculated the odds of concurrent LSIL cytology, vs. a result of normal or reactive changes and we looked at the relationship between vaginal pH and high grade lesions(HSIL) compared to ≤ LSIL among HPV positive women.

When appropriate to increase statistical power and stability of estimates, we included vaginal pH as a dichotomous variable (pH < 5.0 and pH ≥ 5.0). We used logistic regression to examine the association between vaginal pH and *C. trachomatis *at enrollment. Analyses were performed using Stata 11.0 analytic software (Stata Corp LP, College Station, TX).

## Results

Nine thousand one hundred sixty-five women were included in this study, with a total of 28,915 visit data points. Overall, the mean age at the time of visit was 41.8 ± 15 years (range 18-100 years).

**Figure 2:**
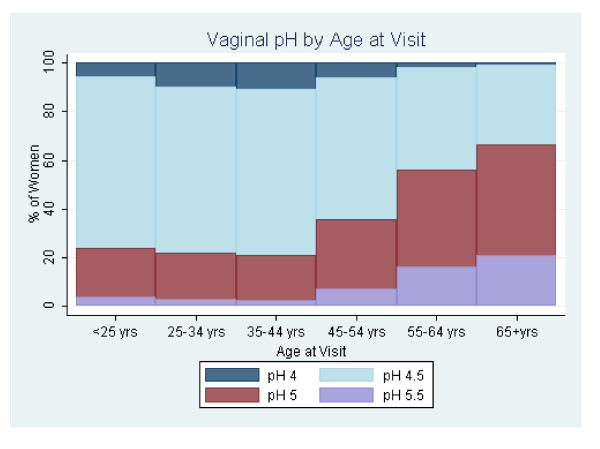
**The percentage of women with a given pH measurement (4.0, 4.5, 5.0, or 5.5) stratified by age at visit**.

Ordinal logistic regression analysis revealed a significant relationship between vaginal pH and age at visit (OR 1.6 95% CI 1.6-1.6). Figure 2 shows the percentage of women with a given pH measurement for each age group at the time of visit. The majority of women younger than 45 (age groups < 25 years, 25-34 years, and 35-44 years) had a pH of 4.5 (~68% for each age group) while a relatively low percentage had a pH of 5.5 (~3.0%). On the other hand, almost half of the women 55 and older (age groups 55-64 years and 65+ years) had a pH of at least 5.0 and a relatively higher percentage of these age groups compared to younger women had a pH of 5.5 (~16% and 21% for women 55-64 and 65+ years, respectively).

The prevalence of HPV was highest in the 25-34 year age group and decreased somewhat steadily among the older age groups, with a slight increase in women aged 65+ years in all visit data (data not shown).

Detection of HPV was positively associated with vaginal pH in women in all age groups under age 55 with about a 10-20% increased risk of HPV detection for a vaginal pH ≥ 5 (Table 1). At the post-menopausal age ranges (55-64, and 65+ years, respectively), there were no significant associations between vaginal pH and detection of HPV. The data were underpowered to conduct a type-specific analysis; however, we assessed at the association between vaginal pH and a pool of 12 carcinogenic HPV types. The results were similar to those in Table 1 with the exception of women aged 65+, who showed a stronger, significant association between a vaginal pH > 5.0 and carcinogenic HPV positivity (GEE OR 1.6 95% CI 1.1-2.3).

**Table 1:** Risk of testing PCR positive for HPV at each visit given vaginal pH measurement, stratified by age at visit

Vaginal pH Level	N	Percent with PCR Positive HPV	Odds of Testing HPV Positive^a ^(95% CI)	*p*-value (trend)
All Women^b^				
4.0	2,139	19.1	0.7 (0.7-0.8)	
4.5	17,201	23.8	ref^c^	
5.0	7,246	25.6	1.2 (1.1-1.3)	
5.5	1,976	25.2	1.2 (1.1-1.4)	< 0.001

Age at Visit				
< 25 years				
4.0	114	22.8	0.5 (0.3-0.8)	
4.5	1,417	38.3	ref	
5.0	404	40.8	1.1 (0.9-1.4)	0.009
5.5	82	41.5	1.1 (0.7-1.8)	

25-34 years				
4.0	798	22.7	0.8 (0.7-1.0)	
4.5	5,436	26.5	ref	
5.0	1,517	29.8	1.2 (1.0-1.3)	
5.5	240	29.2	1.1 (0.9-1.5)	0.007

35-44 years				
4.0	842	17.5	0.9 (0.7-1.1)	
4.5	5,244	19.6	ref	
5.0	1,407	21.4	1.1 (1.0-1.3)	
5.5	194	16.0	0.8 (0.5-1.1)	0.088

45-54 years				
4.0	310	13.6	0.8 (0.5-1.1)	
4.5	2,912	17.2	ref	
5.0	1,432	19.8	1.2 (1.0-1.4)	
5.5	372	18.0	1.1 (0.8-1.4)	0.051

55-64 years				
4.0	48	12.5	0.4 (0.2-1.0)	
4.5	1,253	25.1	ref	
5.0	1,194	23.3	0.9 (0.7-1.1)	
5.5	481	23.9	0.9 (0.7-1.2)	0.209

65+ years				
4.0	27	22.2	0.7 (0.3-1.9)	
4.5	939	27.7	ref	
5.0	1,292	29.1	1.1 (0.9-1.3)	
5.5	607	29.7	1.1 (0.8-1.4)	0.773

^a ^Generalized estimating equation models with an independent correlation structure were used to estimate the odds of testing positive for HPV given pH measurements at each visit

^b ^Adjusted for age at visit

^c ^A pH of 4.5 was used as the referent category because it provided the most stable comparison

A similar positive association was found between elevated vaginal pH (5.0-5.5) and detection of more than one HPV type among all women (HPV positive and negative) (GEE OR 1.3; 95% CI 1.1-1.4) when adjusted for age at visit (Table 2). Age-stratified analyses revealed significant, positive associations between vaginal pH and detection of multiple HPV types vs. infection with a single type in women aged < 25 and 25-34 years as they were 50% and 30% more likely to have a multiple infections, respectively. This relationship was also significant among women aged 65+ years (GEE OR 1.6; 95% CI 1.1-2.2).

**Table 2:** Risk of testing PCR positive for multiple HPV types at each visit given vaginal pH measurement, stratified by age at visit

Vaginal pH Level	N	Percent with Multiple HPV Types	Odds of Testing PCR Positive for Multiple HPV Types^a ^(95% CI)	*p*-value
All Women^b^				
4.0-4.5	4,118	35.1	ref	< 0.01
5.0-5.5	2,165	39.4	1.4 (1.1-1.4)	

Age at Visit				
< 25 years				
4.0-4.5	535	46.2	ref	0.01
5.0-5.5	187	56.7	1.5 (1.1-2.1)	

25-34 years				
4.0-4.5	1,501	34.6	ref	0.01
5.0-5.5	483	41.2	1.3 (1.1-1.6)	

35-44 years				
4.0-4.5	1,053	30.4	ref	0.42
5.0-5.5	307	32.9	1.1 (0.8-1.5)	

45-54 years				
4.0-4.5	492	32.1	ref	0.48
5.0-5.5	314	29.6	0.9 (0.6-1.2)	

55-64 years				
4.0-4.5	297	38.7	ref	0.16
5.0-5.5	365	32.9	0.8 (0.5-1.1)	

65+ years				
4.0-4.5	240	35.0	ref	< 0.01
5.0-5.5	509	46.0	1.6 (1.1-2.2)	

^a ^Generalized estimating equation models with an independent correlation structure were used to estimate the odds of testing positive for multiple HPV given dichotomous pH measurements at each visit

^b ^Adjusted for age at visit

When adjusted for age at visit, a vaginal pH of ≥ 5.0 was positively associated with a diagnosis of LSIL vs. normal cytology including reactive changes (GEE OR 1.3; 95% CI 1.1-1.6) (Table 3). This positive relationship was present in all age groups, and was significant in women < 25 and 25-34 years of age (GEE OR 1.5 95% CI 0.9-2.3; GEE OR 1.4 95% CI 1.0-1.9, respectively) and in women 65+ years of age (GEE OR 2.7 95% CI 1.0-7.0).

**Table 3:** Risk of low-grade squamous intraepithelial neoplasia (LSIL) diagnosis at each visit given vaginal pH measurement, stratified by age at visit

Vaginal pH Level	N	Percent with LSIL	Odds of LSIL Cytology^a ^(95% CI)	*p*-value
All Women^b^				
4.0-4.5	19,272	2.4	ref	< 0.01
5.0-5.5	9,059	2.5	1.3 (1.1-1.6)	

Age at Visit				
< 25 years				
4.0-4.5	1,536	4.0	ref	0.12
5.0-5.5	475	5.7	1.5 (0.9-2.3)	

25-34 years				
4.0-4.5	6,178	2.9	ref	0.02
5.0-5.5	1,721	4.0	1.4 (1.0-1.9)	

35-44 years				
4.0-4.5	6,074	2.3	ref	0.30
5.0-5.5	1,568	2.8	1.2 (0.9-1.7)	

45-54 years				
4.0-4.5	3,216	1.9	ref	0.33
5.0-5.5	1,776	2.3	1.2 (0.8-1.8)	

55-64 years				
4.0-4.5	1,308	1.3	ref	0.82
5.0-5.5	1,653	1.4	1.1 (0.6-1.9)	

65+ years				
4.0-4.5	960	0.5	ref	0.04
5.0-5.5	1,866	1.4	2.7 (1.0-7.0)	

^a ^Generalized estimating equation models with an independent correlation structure were used to estimate the odds of testing positive LSIL given dichotomous pH measurements at each visit

^b ^Adjusted for age at visit

To examine the question of whether elevated pH might be associated with progression to precancer among HPV infected women, we restricted our analyses to HPV positive women but found no association between HSIL and elevated pH (data not shown).

To assess whether vaginal pH could reflect (or predict) the presence of other sexually transmitted infections, we analyzed the relationship between the presence of *C. trachomatis *DNA detected in cervical exfoliated cells and vaginal pH at enrollment in the subgroup of women previously described. Among women < 25 years, we found a positive association between vaginal pH and presence of *C. trachomatis *DNA (OR 2.2 95% CI 1.0-5.0), with women with a pH of > 5.0 (n = 37) more likely to have a *C. trachomatis *infection (32.4%) versus women with a pH of < 5.0 (n = 135), of whom only 17.8% were positive (data not shown). This positive relationship was present among women younger than 45 years, however in women aged 45+, an inverse trend was observed, though these results were not significant.

## Discussion

We report a positive association between vaginal pH and HPV positivity in a very large cohort of randomly selected women. The relationship between HPV and vaginal pH was particularly pronounced in pre-menopausal women (< 25 and 25-34 years). The presence of multiple HPV type infections was also significantly related to elevated vaginal pH overall and particularly in women under 35 years and in women aged 65+ years. In line with these results, we also found a significant positive association between increased vaginal pH and LSIL, a cytomorphic indicator of HPV infection. This relationship was particularly significant in the youngest (< 25 and 25-34 years) and the oldest (65+ years) age groups.

Our study confirmed that vaginal pH rises with age, starting in the peri-menopausal age range. These results are in line with a previous cross-sectional study evaluating the epidemiologic determinants of vaginal pH in the same-population based sample from Guanacaste (enrollment data only) in which vaginal pH was strongly related to age and menopausal status [23]. However the authors did not find an association between elevated pH and HPV infection (11 carcinogenic types) in the baseline data [23].

HPV is a sexually transmitted infection and the association of HPV prevalence with elevated vaginal pH could be due to some unmeasured aspect of sexual behavior. However, changes in the vaginal microenvironment associated with pH in younger women affect the immunological balance within the cervical tissue and provide a biologically plausible mechanism for increased HPV prevalence [24,25]. A healthy vaginal microflora in women of reproductive age maintain a low vaginal pH (< 4.5) [8]. A high vaginal pH is indicative of changes within the vaginal microflora and is used as a criterion in the diagnosis of BV [8] in which several types of anaerobic bacteria, such as *Gardernella vaginalis*, predominate in the vaginal cavity [26]. The absence of protective lactobacilli and an increased vaginal pH has been shown to increase susceptibility for sexually transmitted infections, and thus t could possibly be associated with elevated risk of HPV acquisition [5]. Interestingly, we also found a positive relationship between *C. trachomatis *DNA and vaginal pH among women < 25 years. Previous *in vitro *studies have demonstrated a significant reduction of *C. trachomatis *replication when exposed to acidic pH [11,27] and a prospective case-control study [28] found that *C. trachomatis *infection was associated with higher vaginal pH even when controlling for BV. Taken altogether, these findings suggest a common link between increased vaginal pH and susceptibility to acquiring sexually transmitted infections, and warrant further exploration.

Although the relationship between elevated vaginal pH and overall HPV positivity was not significant among women aged 65+ years, we did find a positive association between vaginal pH of ≥ 5.0 and detection of carcinogenic HPV, multiple HPV type infections, and diagnosis of LSIL in these women. Risk of HPV infection may be exacerbated by an age-related attenuation of the immune response and reductions of the natural immune defenses of the skin, typical of menopause [29]. The vaginal pH is known to increase after menopause, as circulating estrogens decline and cause a depletion of glycogen and glucose metabolism [8]. Our findings among post-menopausal women may draw a link between the decline in circulating estrogens, increased vaginal pH, and decreased immune competence leading to possible increased acquisition of new HPV infections and/or reactivation of latent infections.

There were several limitations in our study. We realize that we did not have adequate information regarding vaginal conditions such as BV which could have been useful in characterizing underlying causes of elevated vaginal pH. Furthermore, this study did not include data on female and/or male sexual behavior, an especially important determinant of HPV prevalence in women in Guanacaste. Therefore, we were unable to address whether the relationship between vaginal pH and HPV was confounded by sexual activity. Finally, we did not measure pH and HPV frequently enough to assess the temporal direction of any possibly causal relationship. Thus, we are not claiming that the associations we found suggest that elevated pH increases HPV prevalence. This is meant to be the report of a novel epidemiologic association.

## Conclusions

Our data suggest that elevated vaginal pH is related to detection of HPV, particularly multiple type infections, and LSIL in certain age groups and with detection of *C. trachomatis *DNA in women aged < 25 years. Future studies should obtain closely spaced measurements, permitting a focus specifically on the temporal sequence of HPV acquisition and increased vaginal pH, and should include data on pH modulators such as the vaginal microflora, chronic inflammation, and/or pre-and-peri-menopausal hormone levels. If increased vaginal pH is found to precede an HPV infection, this might elevate the clinical significance of measuring vaginal pH. However, utility of this or any biomarker must be firmly established before introduction into clinical practice. In addition to studying vaginal pH, we anticipate that by using high-throughput sequencing of a shared region of bacterial ribosomes (16S ribosomal RNA) [30], we can more precisely define altered vaginal ecosystems and explore the associations of the cervical microbiome as a modifier of HPV natural history in the development of cervical precancer and cancer.

## Competing interests

The authors declare that they have no competing interests.

## Authors' contributions

MC: Analyzed data, drafted manuscript. ACR: Made substantial contributions to conception and design of study and acquisition of data and provided critical review of the manuscript. JG: Contributed substantially to analyses and interpretation of data and provided critical revisions of manuscript. RH: Made substantial contributions to conception and design of study and acquisition of data and provided critical review of the manuscript. AH: Made substantial contributions to conception and design of study and acquisition of data and provided critical review of the manuscript. SW: Biostatistician, involved in the design of the study and provided critical revisions of manuscript. RB: Laboratory collaborator, performed HPV typing, provided critical revisions of manuscript. MS: Principal Investigator, involved in the conception and design of the study and provided critical revisions of manuscript. All authors read and approved the final manuscript.

## Sources of support

National Institutes of Health contracts (N01-CP-21081, N01-CP-33061, N01-CP-40542, N01-CP-50535, N01-CP-81023) and grant (CA78527 to R.D.B.). The Guanacaste cohort (design and conduct of the study, sample collection, cytology, data management, analysis and interpretation of the data) was funded by the Intramural Research Program of the National Cancer Institute, National Institutes of Health, Department of Health and Human Services. HPV testing was supported by Dr. Burk's grant support.

## Pre-publication history

The pre-publication history for this paper can be accessed here:

http://www.biomedcentral.com/1471-2334/12/33/prepub
